# Electric- Field-Modified In Situ Precise Deposition of Electrospun Medical Glue Fibers on the Liver for Rapid Hemostasis

**DOI:** 10.1186/s11671-018-2698-8

**Published:** 2018-09-10

**Authors:** Wei-Ling Luo, Jun Zhang, Xuan Qiu, Li-Juan Chen, Jie Fu, Peng-Yue Hu, Xin Li, Ren-Jie Hu, Yun-Ze Long

**Affiliations:** 1 Collaborative Innovation Center for Nanomaterials and Devices, College of Physics, Qingdao University, Qingdao, 266071 China; 2 Medical College, Qingdao University, Qingdao, 266071 China; 3 Department of Oncology, Qingdao Haici Medical Treatment Group, Qingdao, 266034 China

**Keywords:** Electrospinning, Medical glue fibers, In situ deposition, Liver resection, Rapid hemostasis

## Abstract

Precise deposition of nanofibers is still an important issue in the applications of electrospinning (e-spinning), especially in rapid hemostasis of organs such as the liver, lung, and kidney. In this study, we propose an electric field-modified e-spinning technique with a metal cone attached to the spinning nozzle to realize controllable precise deposition of fibers. The deposition range of the e-spun fibers is tunable by changing the size of the metal cone, and the mechanism is attributed the focused electric field verified by theoretical simulations. This electric field-modified e-spinning method was further used to in situ precisely deposit medical glue *N*-octyl-2-cyanoacrylate (NOCA) fibers onto the resection site of rat liver to realize rapid hemostasis within 10 s. Postoperative pathological results indicate that less inflammatory response and tissue adhesion are observed in this electric field-modified e-spinning group compared with that of traditional airflow-assisted group. This technique combined with our designed handheld e-spinning device could be used in emergency medical treatment, clinics, field survival, and home care for its portability and precise deposition characteristics.

**Electronic supplementary material:**

The online version of this article (10.1186/s11671-018-2698-8) contains supplementary material, which is available to authorized users.

## Background

Liver resection is an effective way to treat cancers in the liver [1]. However, heavy bleeding usually occurs in liver resection due to the abundant blood vessel in this special site [2]. Failure to stop bleeding timely can lead to serious organ failure which could even threaten the human life [3]. Current methods to stop bleeding are mostly focused on mechanical methods like suture and ligation, thermal methods like electrocautery [4], and using hemostatic method agents like fibrin sealants [5, 6], gelatin matrix [7], and chitosan hydrogel adhesive [8]. Of course, all of them have obvious advantages and limitations. For example, suture is the most effective way to stop bleeding, but it needs a timely and meticulous process; otherwise, it causes long-term ischemia [9]. Similarly, thermal methods can damage the local tissues and may make it abnormal from normal tissue which cannot be distinguished easily [10]. Moreover, fibrin sealants widely used for hemostasis can easily lead to adverse human immune response, and they also have disadvantages such as short shelf life, vulnerable to microbial intrusion, and high price [11]. In contrast, e-spinning technology shows excellent potential in hemostasis for its special features such as using less dosage and coating on wound sites even with irregular surfaces [12, 13]. However, the existing e-spinning techniques and devices for hemostasis still have several problems to overcome: (1) volume and weight are so bulky that they cannot be easily carried around, (2) inaccurate deposition of fibers [14] takes a longer time to realize the same hemostasis effect and may also cause tissue adhesion after operation, and (3) they depend on the urban electricity supply, so they are not suitable for usages in outdoor and remote areas without power supply [15]. Although our group recently reported an airflow-assisted e-spinning technique which utilizes an air-pump blower to enable orientated deposition of fibers [12], it needs additional power supply for the air pump. Therefore, a portable e-spinning technique and device that do not rely on mainly electricity but can also achieve orientated deposition of fibers for rapid hemostasis are highly desired.

A metal plate placed in the electrostatic field will generate inductive charges on its surface due to the electrostatic interaction, which can induce a new electric field and thus change original electrostatic field distributions [16–18]. On the other hand, the e-spinning process utilizes the unstable whipping and splitting of charged jets during electrostatic field to achieve micro-/nanofibers and ultimately deposit on a grounded collector [19, 20]. The charged jet is sensitive to the distribution of electrostatic field, so thinner fibers are usually achieved by changing the voltage [21, 22]. Therefore, based on this principle described above, we can introduce a metal plate in the e-spinning process to produce more orientated deposition by decreasing the divergence angle of the flying jet via changing the distribution of the electrostatic field. In addition, we take clinically used cyanoacrylate (CA) medical glue [23] as a hemostasis drug [24], because a large dosage is usually required in clinics to form a thick film for hemostasis. However, this film is rigid for the large thickness of the CA medical glue. On the contrary, polymer fiber membranes generated by e-spinning methods are often flexible and compact enough [25]. Therefore, it is of great significance to use electrostatic field-modified methods for e-spinning CA medical glue with precise deposition on the liver for rapid hemostasis.

In this study, we propose an electric field-modified e-spinning technique to realize controllable precise deposition of medical glue fibers on the liver resection site. The deposition range of the e-spun fibers is tunable by changing the size of the metal cone. This electric field-modified e-spinning method was further used to in situ precisely deposit medical glue *N*-octyl-2-cyanoacrylate (NOCA) fibers onto the resection site of rat liver to realize rapid hemostasis within 10 s. Postoperative pathological results indicate that less inflammatory response and tissue adhesion are observed in this electric field-modified e-spinning group compared with those in the traditional airflow-assisted group. This technique combined with our designed handheld e-spinning device could be used in emergency medical treatment, clinics, field survival, and home care for its portability and precise deposition characteristics.

## Methods

### Materials

Rapid medical adhesive α-cyanoacrylate (CA) which is composed of *N*-octyl-2-cyanoacrylate and medical grade polymethyl methacrylate (PMMA, an additive to increase viscosity) was provided by Guangzhou Baiyun Medical Adhesive Co., Ltd. and used without further purification. Chloral hydrate was purchased from Aladdin, which was diluted into 10% for further anesthesia.

### In Vivo Hemostatic Experiments

The hemostasis experiments after rat liver resection were operated on 40 adult male SD rats weighing 300~350 g. These rats were randomly divided into two groups for in situ airflow-assisted (*n* = 20) and electric field-modified e-spinning (*n* = 20) treatment. Every rat accepted 0.7 ml 10% chloral hydrate before the operation, then a laparotomy, lobe free, and a 50% liver resection, followed by in situ electric field-modified (electrode side length of 2.5 cm, electrode angle of 60°, e-spinning distance of 10 cm, voltage of 10 kV) or airflow-assisted (outlet diameter of 1.2 mm, voltage of 10 kV, flow rate of 120 μl min^−1^, and e-spinning distance of 10 cm) e-spinning NOCA fibers. The whole process occupied about 20 min for each rat. All operating procedures complied with the National College of Animal Experiments Regulations and University Animal Research Committee management regulations.

### Blood Test and Pathological Sectioning

Blood samples were collected by heart puncture on the third and fifth days after operation for white blood cell (WBC) count detection and liver function tests. The rats were euthanized and the lobe was excised on the seventh day after operation, in which the lobe was further fixed in the 4% neutral formalin solution, embedded in paraffin and stained with hematoxylin and eosin (HE).

### Electric Field Simulation

Finite element analysis method was used to simulate the electric field distribution. The geometrical model consists of a power supply of 12 kV, a copper needle attached with a copper cone, and an aluminum collecting plate in air. The parameters of needle length, cone diameter, and receiving distance were set as 3, 5, and 10 cm, respectively.

### Characterization

SEM imaging was carried out on a Hitachi TM-1000 scanning electron microscope. The Fourier transform infrared (FTIR) spectrum was measured on a Nicolet In10 spectrometer to analyze the fibers’ intermolecular structure. An optical microscope (Olympus BX51) was used to find the deposition boundary and evaluate the deposition area. Casio Exilim camera was used to record the in vivo liver resection process.

## Results and Discussion

### Electric Field-Modified E-spinning for Precise Deposition

**Fig. 1:**
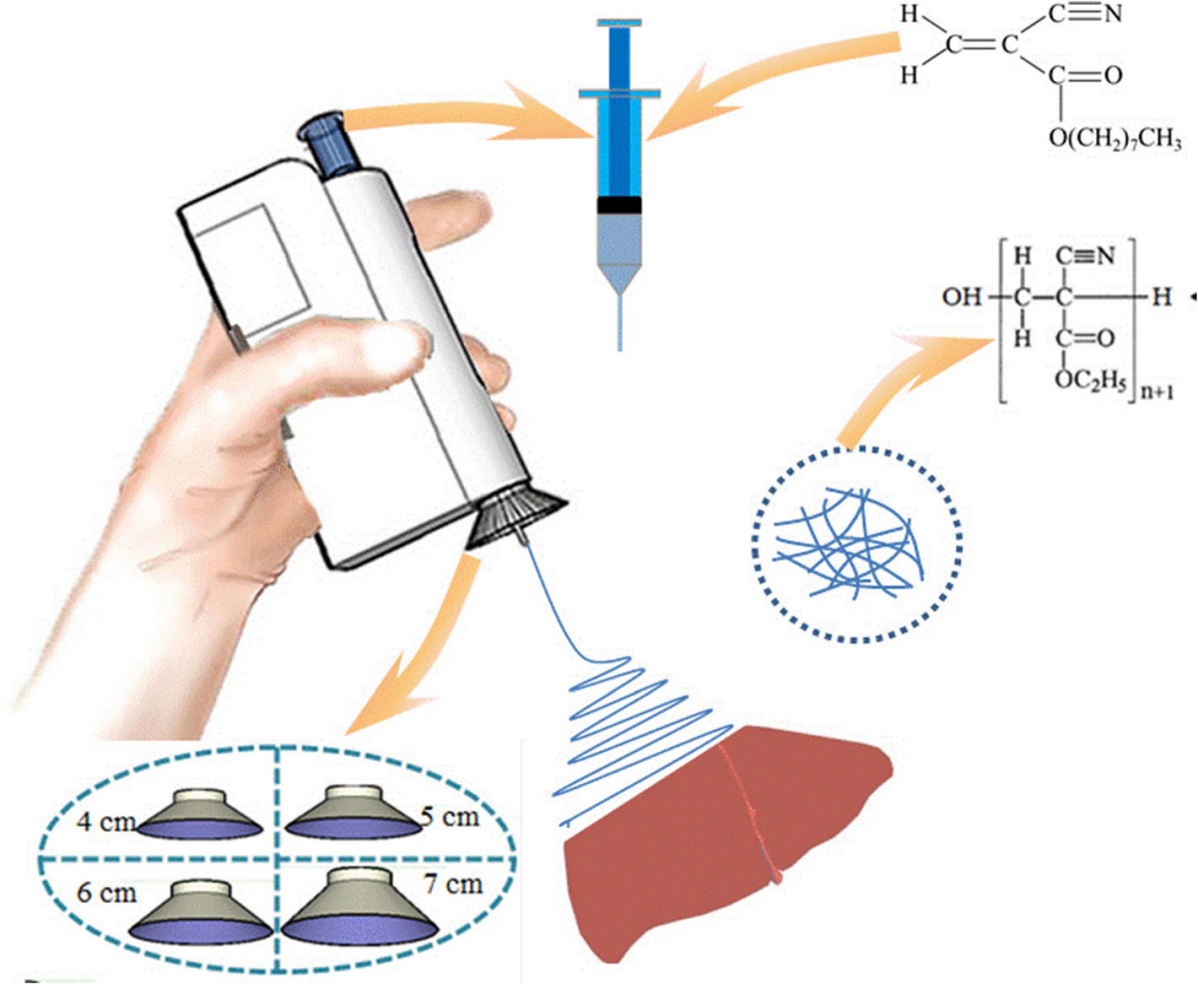
Schematic diagram of the electric field-modified e-spinning NOCA fibers for liver resection hemostasis

Figure 1 and Additional file 1: Figure S1 display our homemade handheld e-spinning device equipped with the electric field-modified e-spinning technique. It uses two mercury-free alkaline AAA batteries (diameter 10 mm, height 44 mm; LR03, Fujian Nanping, Nanfu Battery, China) as power supply with a high-voltage converter and gets rid of the limitation of urban electricity supply that greatly develops the portable use in outdoors. Importantly, significantly different from our recent reported e-spinning device [11], a metallic cone with tunable size is equipped at the spinning needle. The introduction of the metallic cone would change original electromagnetic field distributions and affect the e-spinning process. It should be noticed that the safety issues such as electric shock are usually caused by a high current rather than a high voltage. In this study, the handheld device has a converter which is used to keep a high voltage and a low current to ensure safety.

**Fig. 2:**
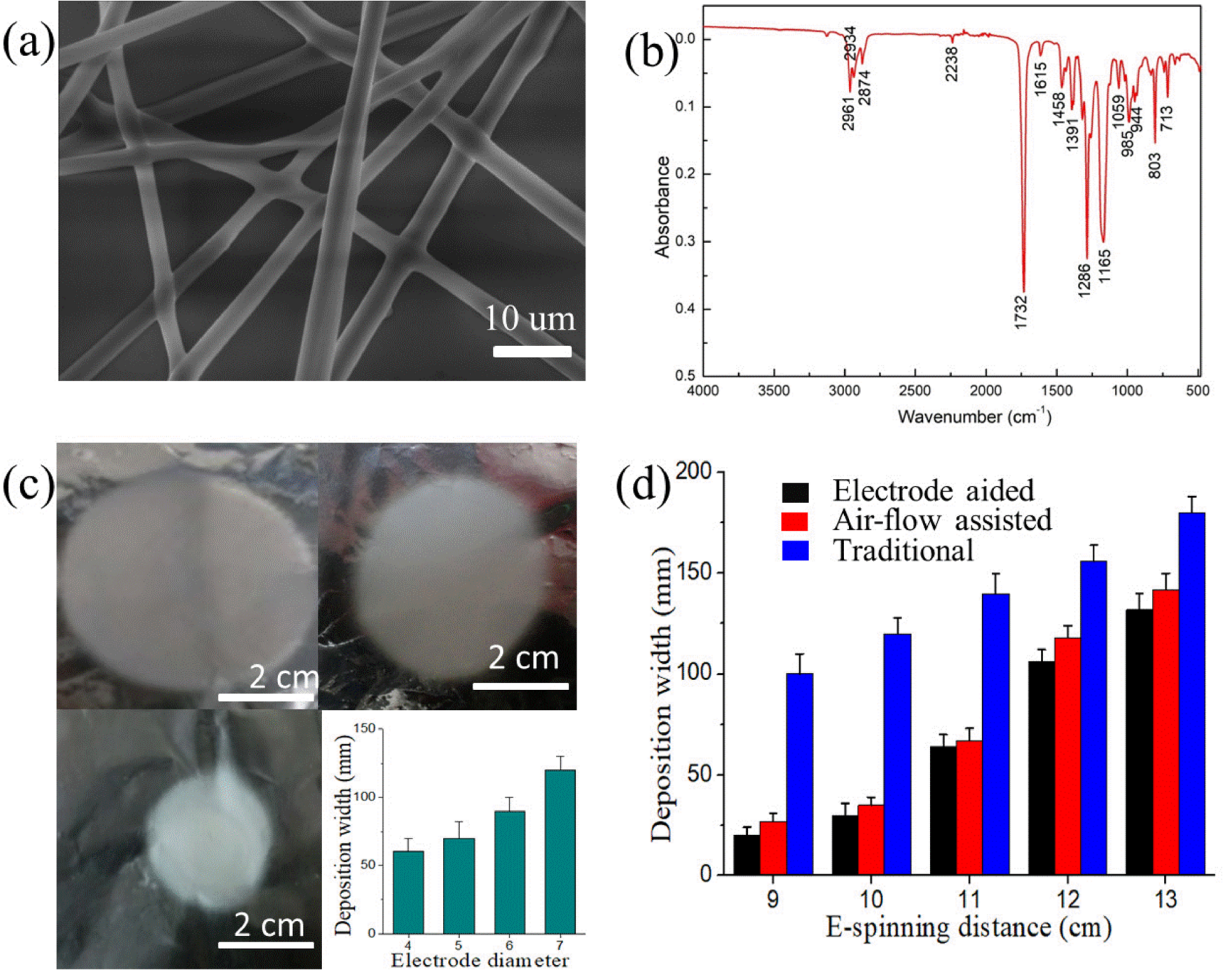
**a** The SEM image and **b** FTIR spectrum of NOCA fibers obtained by the electric-field assisted e-spinning device. The size of deposition area as a function of **c** metallic cone diameter and **d** e-spinning distance

Figure 2a shows the SEM image of NOCA fibers from medical glue. The diameter of the NOCA fibers is about 1~3 μm, and these fibers exhibit a continuous fiber morphology. Figure 2b shows the FTIR spectrum of these NOCA fibers. Peaks at 714 cm^−1^, 2761 cm^−1^, and 1732 cm^−1^ correspond to the vibration of –CH_2_–, –C≡N, and –C=O, respectively. The peak at 3127 cm^−1^ corresponding to =CH– almost disappears, which is caused by the polymerization process during the e-spinning process that most of alkenyl C=C bonds in monomer molecules are transformed to polymer chains. Furthermore, we investigate the relationship between the size of metallic cone and orientated deposition. As shown in Fig. 2c, the diameter of the deposition area decreases with the decreasing of metallic cone size when the distance between the needle tip and the collector was fixed on 10 cm. This phenomenon is probably due to that the electrostatic field would be constrained at a narrower range [26, 27] with decreasing of the metallic cone size, and thus, the whipping process in e-spinning would be more restricted leading to a smaller deposition area. Moreover, the relationship between e-spinning distance and deposition area was also studied (Fig. 2d). Additional file 1: Table S1 presents the deposition width of three different e-spinning methods with increasing e-spinning distance. The concrete deposition found that the deposition area increases with the increase of the e-spinning distance, which is consistent with traditional e-spinning results. However, compared with traditional e-spinning, our electric field-modified e-spinning with a metallic cone brings about a smaller deposition area, namely better orientated deposition. Even compared with our recently reported airflow-assisted e-spinning, this electric field-modified e-spinning exhibits a better orientated deposition. As shown in Fig. 2c, d, tuning e-spinning distance and the side length of the metal cone can focus the electric field and bring about a stronger convergence force. Although some closer part, like the skin or muscle of the abdomen, may produce a force to attract the flying jet, we can tune these two parameters to generate stronger convergence force which can reduce this negative effect from the attraction force. In addition, airflow-assisted e-spinning requires an additional power supply to the air pump, and this field-modified e-spinning can get rid of it, bringing about more convenience.

### Mechanism Analysis of Precise Deposition

**Fig. 3:**
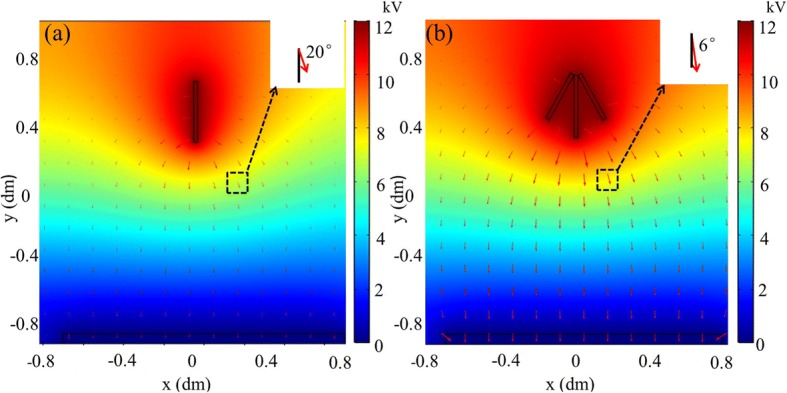
Electric field distribution of e-spinning models equipped **a** without and **b** with a metal cone. Insets are enlarged images of the same area and show the angle between the field line and the vertical direction

To understand the reason why this e-spinning device equipped with a metal cone could bring about a smaller deposition area, their electric field simulations were further conducted. Figure 3 shows the electric field distribution of e-spinning models equipped with and without a metal cone. The red arrow represents the electric field line, whose direction and length stand for the orientation and strength of electric field at this point, respectively. Traditional e-spinning is the one without a metal cone (Fig. 3a), and our electric field-modified e-spinning is the one with a metal cone (Fig. 3b). As shown in Fig. 3, the electric potential (color bar) is significantly decreased along the direction from the needle to the collecting plate, and thus, positive-charged fibers can be assembled on the collecting plate. More interestingly, comparing Fig. 3a with b, stronger electric field strength and smaller divergence angle of electric field direction were observed in Fig. 3b, and these phenomena are more obvious when they are near the metal cone. Its effect on changing the electric field acts like the convergence effect on the light by a convex lens. The electric field lines are convergent, so that it brings about a smaller divergence angle of electric field direction. Moreover, the electric field intensity at the same position also becomes larger due to this convergence and the superposition principle of electric field. The inset is the representative electric field line selected from the same area with magnification. The field strength is 4 × 10^5^ V/m in Fig. 3b inset, which is larger than 3 × 10^5^ V/m in Fig. 3a inset, indicating the larger electric field strength occurs in space after adding a metal cone. And the divergence angle of electric field direction is 6° in Fig. 3b inset, which is smaller than 20° in Fig. 3a inset. These results imply that this electric field-modified e-spinning equipped with a metal cone bringing about a smaller deposition area can be attributed to stronger electric field strength and smaller divergence angle, which constrict the positive-charged fibers flying in a narrower space thereby confining their deposition to a smaller area.

### In Vivo Rapid Hemostasis and Analysis

**Fig. 4:**
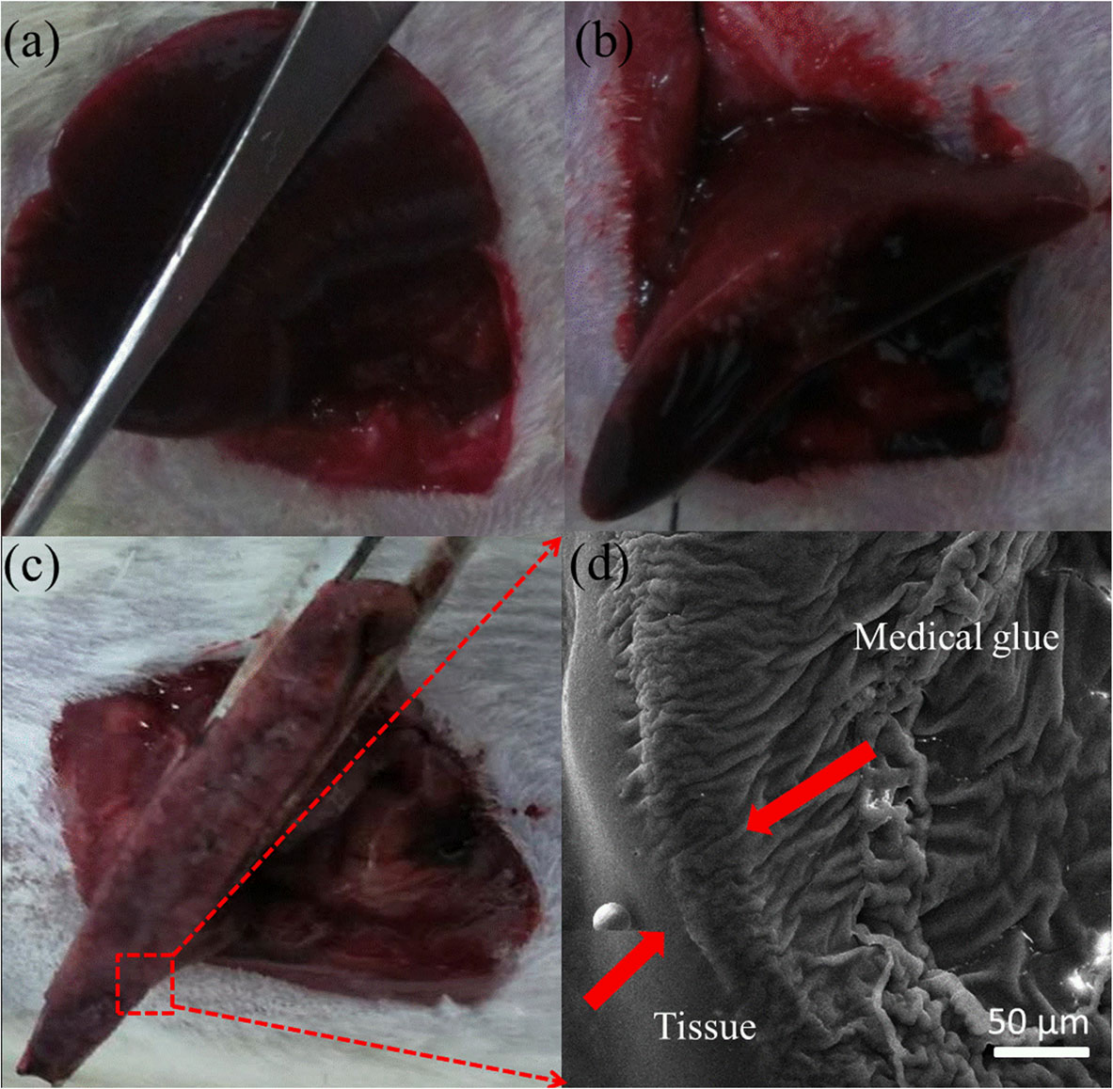
Hemostasis in a rat liver resection model through in situ electric-field assisted e-spinning. **a** The liver was dissociated and the liver lobe was exposed. **b** The lobe was free and fixed with a surgical suture to temporarily block the hepatic blood flow. **c** A hepatectomy was made and NOCA medical glue fibers were deposited on the wound site with our electric-field assisted e-spinning device. **d** Cross-sectional SEM image of NOCA medical glue fibers deposited on the liver surface for hemostasis

Figure 4a–c shows the main process of hemostasis in rat hepatic resection. A rapid and effective hemostasis was achieved within 10 s by NOCA fibers using this electric field-modified e-spinning technique, which is more quick than that of airflow-assisted e-spinning. This phenomenon can be attributed to the better orientated deposition of electric field-modified e-spinning than airflow-assisted e-spinning verified in Fig. 2d, which means the same amount of medical glue can be more accurately deposited on the wound site during the same e-spinning time. In fact, NOCA medical glue used in clinics usually takes a spraying way [28–30], while the deposition area is relatively large leading to some serious tissue adhesions, which makes it difficult to perform postoperative operations such as removal of sutures and even cause secondary damage. Better orientated deposition not only enables faster hemostasis, but also can avoid tissue adhesion. Figure 4d shows the cross-sectional SEM image of NOCA fibers that deposited on the liver surface for hemostasis. It shows that NOCA fibers are tightly adhered to the surface of the liver section and formed a compact fiber membrane whose thickness is about 50 μm with the e-spinning time of 10 s. During this short e-spinning time of 10 s, the distance change caused by the hand shake that usually comes from the fatigue is tiny, usually no more than 1 cm, and thus, the variation of the deposition range is small. More interestingly, the surface of the liver section is not smooth but irregular in shape (Fig. 4c), while NOCA fibers could deposit onto this irregular surface with a good uniform thickness (Fig. 4d), implying that this electric field-modified e-spinning technique possesses unique advantages in rapid hemostasis even on some irregular surfaces of organs.

**Fig. 5:**
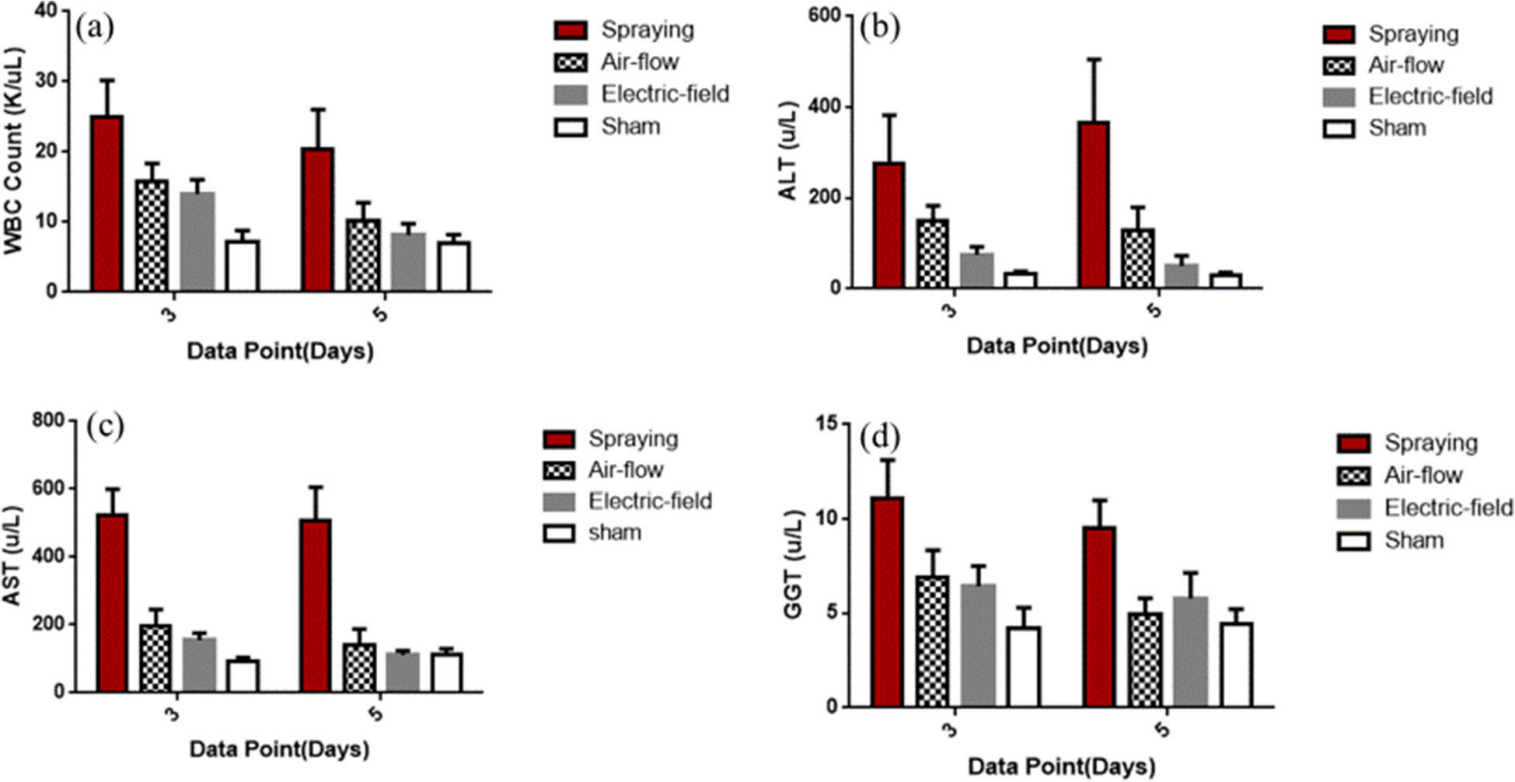
Blood test. **a** WBC count. **b**–**d** Liver function enzyme test. **b** Alanine aminotransferase (ALT). **c** Aspartate aminotransferase (AST). **d** Glutamyltransaminase (GGT)

The WBC count test (Fig. 5a) was used to evaluate postoperative infections caused by hepatectomy and hemostasis in rats. Five days after surgery, the number of WBC (*P* < 0.05) in the electric field-modified e-spinning group was significantly lower than that of the conventional spraying group and the airflow-assisted group (*P* < 0.01). Moreover, it was close to the sham-operated group (control group), which indicates that the acute inflammation after 5 days in the electric field-modified e-spinning group subsided to a normal state. On the contrary, rats in the spraying group and the airflow-assisted group show serious inflammatory response and slower regression.

Liver function was evaluated by the concentration of the serum ALT (Fig. 5b), AST (Fig. 5c), and GGT (Fig. 5d). Herein, ALT and AST concentration can sensitively reflect the extent of liver cell damage. High concentrations of GGT can reflect hepatitis, obstructive jaundice, bile stasis, and other symptoms. As shown in Fig. 5b–d, the liver function enzyme levels in electric field-modified e-spinning group after 5 days of operation were basically close to those in the sham group (control group) and were significantly lower than those in the conventional spraying group and the airflow-assisted group, indicating that the physiological state of the rats in the electric field-modified e-spinning group and the sham group was similar. However, GGT in the spraying group and airflow-assisted group still remained a high level on the fifth day after operation (*P* < 0.001), indicating that there are some serious problems such as bile stasis and liver damage.

**Fig. 6:**
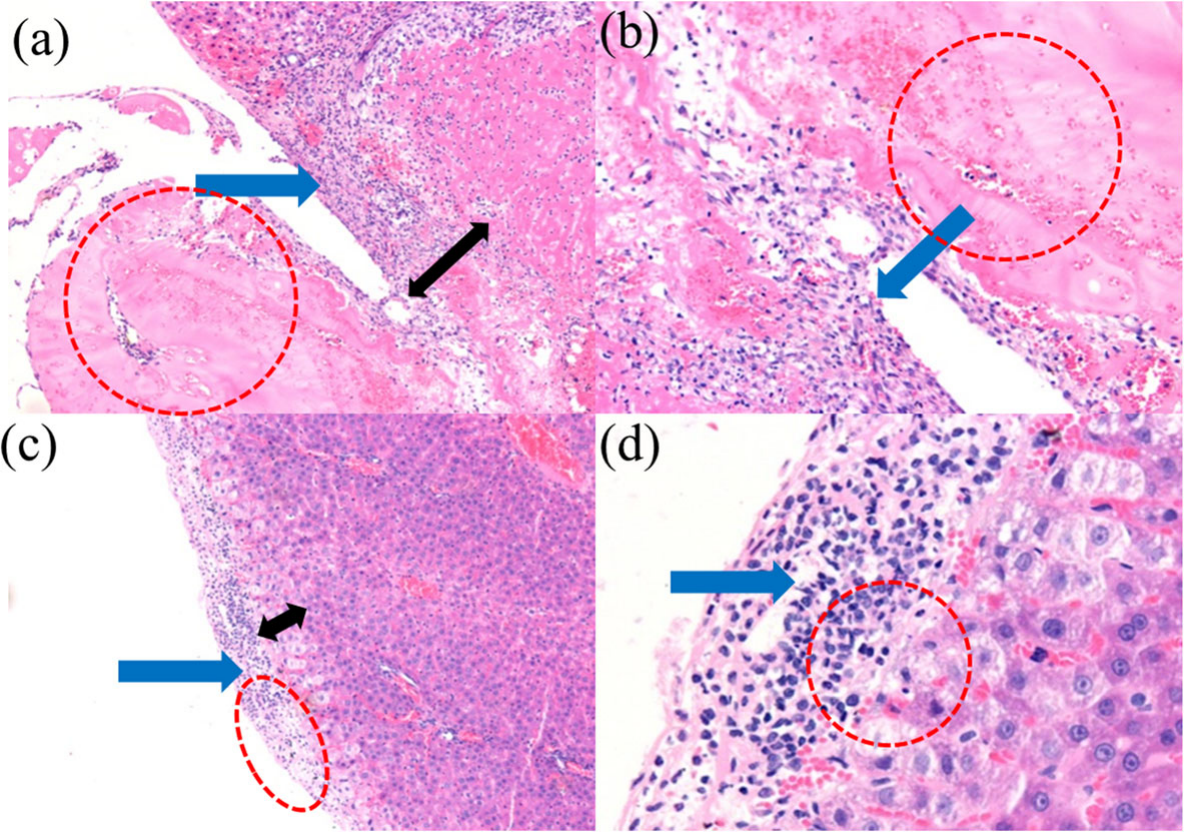
Histopathological examination with HE staining observed under **a**, **c** magnification × 100 and **b**, **d** magnification × 200. Histopathological examination shows an inflammatory response and liver injury among hepatocytes in two groups at the seventh day. The two groups are the **a**, **b** airflow-assisted group and **c**, **d** electric field-modified group (blue arrow: inflammatory cells; red circle: medical glue; black arrow: thickness of hyperemia zone)

The pathological biopsy on liver tissues after hemostasis was further conducted. Figure 6a and c are liver pathological sections after hemostasis with airflow-assisted and electric field-modified e-spinning, respectively, and Fig. 6b and d are their enlarged images. Compared with airflow-assisted e-spinning group, the liver tissue boundaries in the electric field-modified e-spinning group is relatively clearer and has a thinner capsule. These results indicate that the regeneration ability in the liver is better in the electric field-modified group. Moreover, less inflammatory cells were observed in the capsule, indicating that the NOCA fibrous membranes fabricated by the electric field-modified method can bring about less inflammatory response. These results can be attributed to the fact that electric field-modified methods have better orientated deposition than airflow-assisted methods, thus reducing the amount of the NOCA medical glue used for achieving the same hemostatic effect, which will reduce the tissue adhesion and thereby inflammatory response. In addition, it also can be seen from Fig. 6a, b that medical glue was separated from the liver tissue, which may be caused by the air blow, indicating the adhesion between them using airflow-assisted e-spinning is not as strong as the electric field-modified e-spinning.

## Conclusions

In summary, we propose an electric field-modified e-spinning technique with a metal cone attached to the spinning nozzle to realize controllable precise deposition of fibers. The deposition range of the e-spun fibers is tunable by changing the size of the metal cone, and the mechanism is attributed to the focused electric field verified by theoretical simulations. This electric field-modified e-spinning method was further used to in situ precisely deposit medical glue NOCA fibers onto the resection site of rat liver to realize rapid hemostasis within 10 s. Postoperative pathological results indicate that less inflammatory response and tissue adhesion are observed in this electric field-modified e-spinning group compared with that in the traditional airflow-assisted group. This technique combined with our designed handheld e-spinning device could be used in emergency medical treatment, clinics, field survival, and home care for its portability and precise deposition characteristics.

## Additional File

Additional file 1: **Figure S1.** The photograph of our homemade portable handheld e-spinning device equipped with the electric field-modified technique that a metal cone is added to the spinning nozzle. Table S1. Deposition widths of various e-spinning methods at different e-spinning distances. (DOC 64 kb)

## Abbreviations

ALT: Alanine aminotransferase
AST: Aspartate aminotransferase
CA: Cyanoacrylate
E-spinning: Electrospinning
FTIR: Fourier transform infrared
GGT: Glutamyltransaminase
HE: Hematoxylin and eosin
NOCA: *N*-Octyl-2-cyanoacrylate
PMMA: Polymethyl methacrylate
SEM: Scanning electron microscope
WBC: White blood cell
