# Supplementary material for: Electric- Field-Modified In Situ Precise Deposition of Electrospun Medical Glue Fibers on the Liver for Rapid Hemostasis
Source: Nanoscale Res Lett. 2018 Sep 10;13:278. doi: 10.1186/s11671-018-2698-8 (PMC6134859; doi:10.1186/s11671-018-2698-8)
Supplement: Supplementary file 1 — Figure S1. The photograph of our homemade portable handheld e-spinning device equipped with the electric field-modified technique that a metal cone is added to the spinning nozzle. Table S1. Deposition widths of various e-spinning methods at different e-spinning distances. (DOC 64 kb) [file 11671_2018_2698_MOESM1_ESM.doc]

Additional file 1

**Electric-Field Modified *in Situ* Precise Deposition of Electrospun Medical Glue Fibers on** **Liver for** **Rapid Hemostasis**

Wei-Ling Luo^1,†^, Jun Zhang^1,†^, Xuan Qiu ^2^, Li-Juan Chen^3^, Jie Fu^1^, Peng-Yue Hu^1^, Xin Li^1^, Ren-Jie Hu^1^ and Yun-Ze Long^1,*^

^1^Collaborative Innovation Center for Nanomaterials & Devices, College of Physics, Qingdao University, Qingdao 266071, China

^2^Medical College, Qingdao University, Qingdao 266071, China

^3^ Department of Oncology, Qingdao Haici Medical Group, Qingdao 266034, China

^†^These two authors contributed equally to this work.

***Address correspondence to yunze.long@163.com


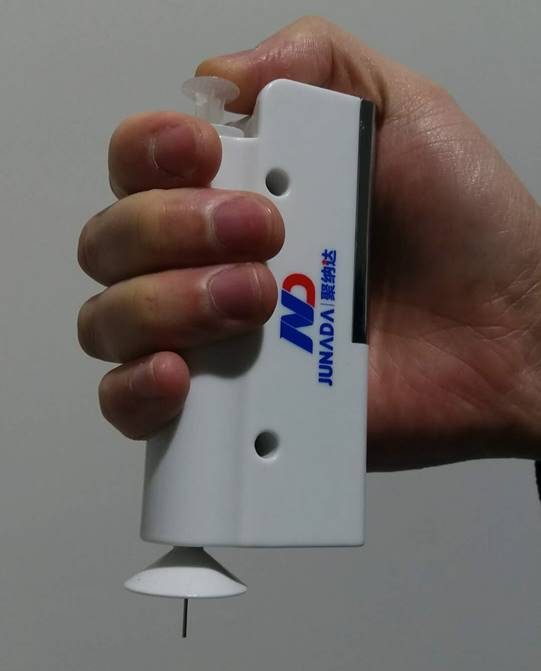


**Figure S1.** The photograph of our home-made portable hand-held e-spinning device equipped with the electric-field modified technique that a metal cone is added to the spinning nozzle.

**Table S1.** Deposition widths of various e-spinning methods at different e-spinning distances.

| E-spinning Distance (cm) | E-spinning Methods | Deposition Width (cm) |
| --- | --- | --- |
| 9 | No Assistance | 104.8±5.5 |
|  | Air-Flow Assisted | 25.6±3.4 |
|  | Electric-Field Modified | 20.6±2.4 |
| 10 | No Assistance | 124±4.6 |
|  | Air-Flow Assisted | 31.8±3.6 |
|  | Electric-Field Modified | 27.3±3.2 |
| 11 | No Assistance | 145.2±4.6 |
|  | Air-Flow Assisted | 67.4±2.8 |
|  | Electric-Field Modified | 61±2.5 |
| 12 | No Assistance | 155.4±3.8 |
|  | Air-Flow Assisted | 116±3.5 |
|  | Electric-Field Modified | 103.8±2.8 |
| 13 | No Assistance | 184.6±4.61 |
|  | Air-Flow Assisted | 137.2±3.03 |
|  | Electric-Field Modified | 130.4±1.14 |
